# Protective effect of hesperetin against myelosuppression and splenic inflammation via modulating TLR4/MYD88/NF-κB signaling: molecular docking and experimental validation

**DOI:** 10.3389/fphar.2026.1826774

**Published:** 2026-08-31

**Authors:** Ghada A. Badawi, Asmaa K. K. Abdelmaogood, Nada Ahmed, Sherihan Rohayem, Shereen Fawzy, Sawsan A. Zaitone, Afaf A. Aldahish, Sozan M. Abdelkhalig, Mohamed A. Helal, Noura Mostafa Mohamed, Aya H. Al-Najjar

**Affiliations:** 1 Department of Pharmacology and Toxicology, Faculty of Pharmacy, Sinai University-Arish Branch, Arish, Egypt; 2 Department of Clinical Pathology, Faculty of Medicine, Suez Canal University, Ismailia, Egypt; 3 Department of Microbiology and Immunology, Faculty of Medicine, Port Said University, Port Said, Egypt; 4 Department of Medical Microbiology and Immunology, Faculty of Medicine, Ain Shams University, Cairo, Egypt; 5 Department of Pharmacology and Toxicology, Faculty of Pharmacy, University of Tabuk, Tabuk, Saudi Arabia; 6 Department of Pharmacology, College of Pharmacy, King Khalid University, Abha, Saudi Arabia; 7 Department of Basic Medical Sciences, College of Medicine, AlMaarefa University, Riyadh, Saudi Arabia; 8 Research Center, Deanship of Scientific Research and Post-Graduate Studies, AlMaarefa University, Riyadh, Saudi Arabia; 9 Biomedical Sciences Program, University of Science and Technology, Zewail City of Science and Technology, 6th of October City, Egypt; 10 Medicinal Chemistry Department, Faculty of Pharmacy, Suez Canal University, Ismailia, Egypt; 11 Department of Basic Sciences, College of Medicine, Princess Nourah bint Abdulrahman University, Riyadh, Saudi Arabia; 12 Pharmacology and Toxicology Department, Faculty of Pharmacy (Girls), Al-Azhar University, Cairo, Egypt

**Keywords:** hesperetin, mouse, myelosuppression, splenic inflammation, TLR4/MyD88/NF-κB, vincristine

## Abstract

**Introduction:**

Myelotoxicity is a common adverse effect of vincristine and other chemotherapeutic drugs. Hesperetin is a bioactive flavanone with anti-inflammatory activity. This study was planned to examine the hesperetin protecting effect against vincristine induced myelosuppression and splenic inflammation in mice and test whether this effect is mediated through the suppression of Toll-like receptor 4 (TLR4)/myeloid differentiation primary response-88 (MYD88)/nuclear factor-kappa B (NF-κB) signaling pathways.

**Methods:**

Bioinformatics tools were used to establish the rationale of the study and molecular docking defined the potential binding of hesperetin to TLR4. Mice were allocated into different experimental groups: Group 1: was the saline mice, Group 2; mice were injected with vincristine (0.1 mg/kg, i. p) for 5 days per week, Group 3; mice received vincristine (as in Group 2) plus hesperetin (100 mg/kg, p. o.) and Group 4; mice received hesperetin (100 mg/kg, p. o.) only; the experiment continued for 2 weeks.

**Results:**

The mouse experiment showed that vincristine produced significant decreases in hematological parameters (mainly leukocytes dropped to 4.6 ± 0.32 and platelets dropped to 799.6 ± 25.46 versus 9.48 ± 0.19 and 1654 ± 273.26 × 10 ^˄^9/L in the saline group, p less than 0.05) and bone marrow hypocellularity (dropped to ∼30–40% compared to 95%–100% in the saline group). The spleens showed white pulp lymphoid depletion, red pulp mild hypocellularity, greater immunostaining for MYD88, and greater content for TLR4, NF-κB, MYD88, TNF-α and IL-1β proteins. Results of molecular docking showed that hesperetin is an interesting complementarity with the TLR4/MD2 interface which was a good rationale for the experimental validation. The mouse experiment showed that hesperetin prevented the decrease hematological parameters, enhanced the bone marrow cellularity to 80%–90%, and the splenic architecture. Hesperetin further inhibited the increases in the target proteins (TLR4, NF-κB, MYD88, TNF-α and IL-1β) significantly.

**Discussion:**

The present data documents the novel role of hesperetin in mitigating myelotoxicity of vincristine through modulation of TLR4/MYD88/NF-κB signaling and a direct binding to TLR4 was proposed by molecular docking. This highlights possible usefulness of this combination after adequate clinical studies.

## 1 Introduction

Myelotoxicity is a common side effect of anticancer drugs due to a decrease in hematopoietic cells production, including leukocytes, erythrocytes, and thrombocytes. This phenomenon leads to the development of various hematological disorders, collectively termed blood dyscrasias, which encompass agranulocytosis, neutropenia, pancytopenia, thrombocytopenia, and aplastic anemia (Gwaltney-Brant, 2019; Tzankov, 2021). Myelosuppression constitutes a significant adverse effect associated with pharmacological agents such as the chemotherapeutic drugs (Daniel and Crawford, 2006; Dessalegn et al., 2023).

Vincristine is a semi-synthetic anticancer drug derived from terpenoid indole alkaloids extracted from Catharanthus roseus (vinca plant) (Waterhouse et al., 2005; Alam et al., 2017). It serves as a cornerstone of therapy in numerous chemotherapeutic regimens for acute and chronic leukemias, neuroblastoma, lymphoma, brain tumors, and thyroid cancer (Bhagat et al., 2024; Shah et al., 2025).

The core problems chalenged vincristine are the diverse side effects including peripheral neuropathy, toxicity to the gastrointestinal tract, vascular disorders and myelosuppression. Vincristine myelosuppression manifestted as inhibiting bone marrow function, primarily decreasing white blood cells and platelets, leading to risks of infetion, fever, bleeding, anemia and bruising (Herradón et al., 2021; Awosika et al., 2023; Bianchini, 2025; Chen et al., 2025). Although vincristine myelotoxicity is typically pericipitated as a mild to moderate side effect, it requires monitoring and management. Importantly, myelotoxicity may attribute to that the appoptotic action of vincristine is not limited to malignant cells but involves impairment of the proliferation of all rapidly dividing cells and affection of leukocyte maturation and production, particularly agranulocytosis (Awosika et al., 2023). Moreover, vincristine interferes with the proliferation and hematopoietic progenitor cell differentiation through macrophage-derived prostaglandins, causing myelosuppression (Jiang et al., 2008). Therefore, strategies that may abbrogate vincristine’s myelotoxicity are essential.

Although the precise mechanisms underlying vincristine-induced bone marrow toxicity remain unclear, the rapid proliferation of hematopoietic cells likely amplifies their vulnerability to its toxicity. Vincristine endorses pro-inflammatory cytokines release such as interleukin (IL) -1β, IL-6, TGF-β as well as the tumor necrosis factor-α (TNF-α) (Wong et al., 2014; Puşcaşu et al., 2024; Chen et al., 2025). The inflammatory milieu may activate toll-like receptor 4 (TLR4), stimulating the myeloid differentiation primary response-88 (MYD88)-dependent and -independent signaling cascades (Janssens and Beyaert, 2002; Ohnishi et al., 2009). Importantly, thrombocytopenia can be induced by TLR activation, specifically via TLR4 (Aslam et al., 2006). Enhanced innate immune signaling, especially TLR signaling, is common in myelodysplastic syndromes. The etiology of the activated TLR signaling may be multifactorial and includes upregulated expression of TLRs and enhanced sensitivity to TLR stimulation (Paracatu and Schuettpelz, 2020). Further, MYD88 is known to have a major influence on the function of the hematopoietic stem cells. One previous study demonstrated that MYD88 activation is an important factor induced myelosuppression (Zhang et al., 2016). Wang et al. (2011) showed that thrombocytopenia pathogenesis is linked to platelet TLR4 expression in (Wang et al., 2011).

Importantly, TLR4–MYD88 signaling has emerged as a shared pathway responsible for various chemotherapy-related side effects, most notably myelosuppression (Ji et al., 2022). Interestingly, TLR4 ligand triggers nuclear factor-kappa B (NF-κB) activation. These insights have prompted the design of therapeutic regimens combining chemotherapy with TLR4 antagonists to prevent drug toxicity (Shetab Boushehri and Lamprecht, 2018; Babolmorad et al., 2021; Liang et al., 2025). The NF-κB transcription factor is the primary downstream mediator of TLR4 pathway, activating a wide array of pro-inflammatory cytokine genes. Therefore, the incorporation of TLR4-directed inhibitory therapy alongside current treatment approaches marks a major leap in precision oncology, enabling reduced side effects by precisely modulating key signaling pathways (Kawai and Akira, 2007; Liu et al., 2025). Thereby, an ideal therapeutic agent would possess anti-inflammatory activity, specifically TLR4 inhibiting activity.

Hesperetin, a flavanone commonly found in citrus juices, is known for its potent antioxidant, anti-inflammatory and antiapoptotic actions suggesting its function as a modulator for anticancer drug toxicities (Roohbakhsh et al., 2015; Sohel et al., 2022). It remains unknown if hesperetin may be able to mitigate drug induced myelosuppression. Since TLR4–MYD88 signaling has emerged as a shared pathway responsible for various chemotherapy-related side effects, studying the possibility that hesperetin may suppress this signaling pathway can be a rational target that has not been previously explored. In previous studies and animal models, hesperetin has been shown to offer neuroprotection via mitigating Nrf2/TLR4/NF-κB signalling (Ren et al., 2016; Ikram et al., 2019).

No previous studies examined hesperetin against vincristine-induced myelosuppression and via the TLR4/MYD88/NF-κB axis. This study explored the protecting effect of hesperetin against vincristine induced myelosuppression and splenic inflammation and to test whether this effect is facilitated through the binding to or suppression of TLR4 as well as the downstream signaling, MYD88/NF-κB pathways. This effect was studied in a multi-evidence design by bioinformatic tools and molecular docking beside the mouse experimental validation study. The intervention-model-pathway combination is first-in-context.

## 2 Materials and methods

### 2.1 The use of bioinformatics tools

Bioinformatics data mining was performed to establish a research rationale for this novel approach to the use of hesperetin, a plant-derived polyphenol, to alleviate myelosuppression induced by a chemotherapeutic agent, Vincristine. TLR4/MYD88/NF-κB pathway was first explored through the KEGG database (www.genome.jp/kegg/) to visualize the target proteins involved in its signalling network (Kanehisa et al., 2023).

STRING database (https://stringdb.org/) was utilized to analyse network interactions of the pathway genes and identify top interactors with TLR4 with a interaction confidence score >0.900 (highest interval available) and to later perform KEGG analysis on the intersecting genes to identify any pathways they contribute to and their biological linkage (Szklarczyk et al., 2021). This approach would enable a more comprehensive insight into protein/protein interactions and how they relate to the central research question. The putative gene/proteins were extracted and tabulated in order to be compared with the predicted targets of hesperetin.

### 2.2 Exploration of predicted targets of hesperetin

We used the PubChem (https://pubchem.ncbi.nlm.nih.gov/; *release 2025.04.14*) for exploring the chemical composition of hesperetin and download its 2D stereochemical structure and SMILES to serve as input for other downstream datasets to accurately explore its predicted targets (Kim et al., 2023).

The Swiss Target Prediction tool of the Swiss institute of Bioinformatics (http://www.swisstargetprediction.ch/) was utilized to explore those targets. Further, SuperPred (https://prediction.charite.de/) was utilized as it enabled computational prediction of putative drug targets for small molecules (Nickel et al., 2014). Finally, we used the PharmMapper platform, V2010 (https://lilab-ecust.cn/pharmmapper/index.html), for identifying the target candidates according to the SDF format file of hesperetin and using only human protein targets for pharmacophore mapping (Wang et al., 2017).

### 2.3 The molecular docking of hesperetin to TLR4

To investigate the mode of binding of hesperetin to TLR4, the docking of hesperetin was done into the experimental structure of TLR4/MD2/LPS complex (PDB ID:3FXI) which was obtained through www.rcsb.org. We employed the FRED section as implemented within the OpenEye 2023.2.3 which uses a rigid docking algorithm (Saeedi et al., 2026).

To validate the molecular docking results, we redocked the LPS complexed with the TLR4/MD2 in their ternary crystal structure (PDB ID: 3FXI) using the same protocol in the FRED module. We used the same conformation of the crystallized LPS without conformational search and the same design unit of the TLR4 receptor (receptor grids) which used after that for hesperetin docking. The software was able to reproduce the binding pose of LPS with adequate similarity to the crystal structure, with an RMSD of 1.205 Å to the crystallized LPS, and a docking score of – 14.895.

Moreover, selected small-molecule TLR4 inhibitors from natural sources, epigallocatechin gallate, curcumin, alpinetin, celastrol, were docked into the same crystal structure of LPS binding site. Their 2D structures were downloaded from PubChem in SDF format and used to generate a conformer library using the defaulting settings of the Omega function of OpenEye. After that, we generated the design unit of the TLR4 receptor (receptor grids) utilizing the graphical interface of the make-receptor module. Docking procedures were currently performed into a box identified via the crystallized *E. coli* LPS. As the last step, the rigid docking was done utilizing the Omega-generated database of ligand confirmers (McGann, 2011).

The generated docking poses were ranked utilizing the default scoring of Chemgauss4 relying on the shape characteristics. This scoring function is composed of the following intermolecular interactions: steric, lone pairs, coordinating groups, donors, acceptor, metals, polar hydrogens and groups coordinating chelation. Pymol 2.5.5 software was used for the preparation of the ligand-protein interaction figures. Finally, hesperetin structure, also downloaded in SDF format from PubChem, was docked according to the previous protocol.

### 2.4 The mouse study

Twenty-eight male albino mice weighing 24 ± 4 g were obtained from Mostafa Rashed Company. Mice were randomized and housed in clean polyethylene cages, as 7 mice per cage. Mice were acclimatized to the study conditions for 5 days before commencing of doses. Mice were kept under a normal day cycle with permitted access to food and water. Animal handling was done by an experienced technician; he was accountable for drug doses and food availability. The study protocol received approval (SU-SREC-5-02-24) by the Research Ethics Committee at the Faculty of Pharmacy, Sinai University. The animal welfare was guided by the principles of the Declaration of Helsinki, Belmont Report and the International Guiding Principles for Biomedical Research Involving Animals developed by the Council for International Organizations of Medical Sciences (CIOMS).

#### 2.4.1 Chemicals and drugs

Hesperetin (Sigma-Aldrich Company, United States of America) was suspended in 1% carboxymethylcellulose (CMC) solution for oral administration. Vincristine sulfate 2-mg ampoules were purchased from EMIC United Pharmaceuticals (Badr City, Cairo, Egypt) and diluted with normal saline to reach the desired concentration.

#### 2.4.2 The experimental design of the mouse study

**FIGURE 1:**
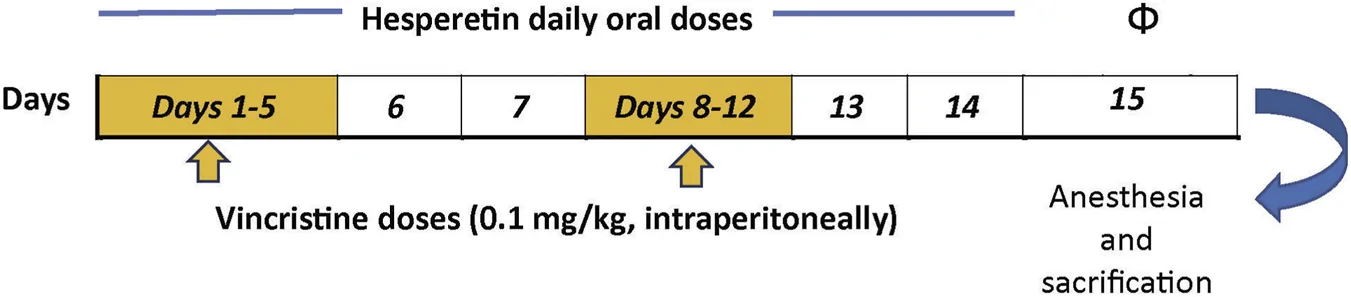
A scheme for study design showing the drug regimen.

Twenty eight male Swiss albino mice were allocated in a random manner into four groups (*n in each group* = 7) using computer-generated random numbers by EXCEL RAND function. The number of mice per group was determined according to similar previous studies (Nawaz et al., 2018; Chen et al., 2020) and assigned as follow (Figure 1):

Group I; the saline control group: mice received saline solution by intraperitoneal injections (i.p.) at a volume of 8 mL/kg at 11 a.m. for five consecutive days in each week. Further, mice received oral doses of 1% CMC solution (parallel to hesperetin doses) at 3 p.m.
Group II; Mice received vincristine sulfate solution (0.1 mg/kg, i. p) in a volume of 8 mL/kg at 11 a.m. for five consecutive days weekly; this regimen was selected based on previous literature (Nawaz et al., 2018; Chen et al., 2020). Further, mice received oral doses of 1% CMC solution (parallel to hesperetin doses) at 3 p.m. The intraperitoneal route is usually preferred in studies examining vincristine toxicity (Harrison Jr, 1983; Ohuchida et al., 1989; Voorhorst et al., 2014).
Group III: Mice received vincristine (the same as Group II) along with daily hesperetin doses (100 mg/kg, p. o.) (Ateyya et al., 2024) at 3 p.m.
Group IV: Mice received saline (i.p., 8 mL/kg at 11 a.m. for five consecutive days in each week) along with daily hesperetin doses (100 mg/kg, p. o.) at 3 p.m. All the treatments continued for a 2-week interval.

#### 2.4.3 Blood collection and preparation of serum samples

After completing the drug schedules, the mice were euthanized using an intraperitoneal dose of ketamine/xylazine mixture (90 and 10 mg/kg). After a midline incision, collection of blood samples was done by the cardiac puncture method. For the complete blood count (CBC), blood samples (1 mL) were transferred to a tube filled with ethylenediaminetetraacetic acid solution (29 mg/mL blood). An automated cell counter (Automated Hematology Analyzer XN series XN-10, Sysmex Corporation, Kobe 651-0073, Japan) was used to analyze blood samples within 2 h (Bilasy et al., 2015). All the analysis procedures were performed blindly.

#### 2.4.4 Microscopic examination of the bone marrow

An experienced clinical pathologist collected the smears from the bone marrow specimens -collected from the femur bones-to be stained with Giemsa stain (Ali, 2015). The pathologist evaluated the bone marrow specimens for the overall cellularity and morphology at differential counts of a minimal of 500 nucleated cells per slide and calculated the ratio of each cell type divided by the total nucleated cells in addition to the myeloid-to-erythroid ratios (M/E). Moreover, megakaryocytes were assessed for morphology and cellularity (Ali, 2015; Bilasy et al., 2015). Microscopic examination was done in a blinded manner.

#### 2.4.5 Processing of the spleen for histopathological examination

To assess the effect of vincristine on hematopoiesis, the spleens and bone marrow samples were used similar to a previous rodent study (Bilasy et al., 2015). The spleen histopathology is considered as the main screening method for assessment of immunotoxicity. The spleens were detached, cleaned with cold saline and weighed. After that, spleens were fixed in 10% paraformaldehyde solution. Then, a specimen was embedded in liquid paraffin for making a paraffin block. Later, a microtome was used to prepare a 4-μm thickness specimen which was then stained with hematoxylin and eosin (H&E) and investigation was done using a light microscope (CX23LEDRFS1, Evident Corporation, Nagando Tokyo, Japan).

We evaluated the spleen for the following: lymphoid follicles cellularity of periarteriolar lymphoid sheaths (PALS), red pulp and the total germinal centers (GCs) count and marginal zone (Bilasy et al., 2015). The scoring system involved ordinal sets starting from 0 (when no effect was observed) up to 4 (when severe effects were observed) (Germolec et al., 2004). The total score was averaged for each group. Microscopic examination and scoring were done in a blinded manner.

#### 2.4.6 Immunohistochemistry for MYD88 in spleen specimens

MYD88 was selected for IHC because it is a key adaptor protein directly downstream TLR4 and plays a central role in mediating TLR4-dependent signaling. Assessment of MYD88 provides evidence regarding the activation status of the TLR4/MYD88 axis in the early stage of the pathway. Using specimens from the mice spleens, immunohistochemical staining for MYD88 was performed. 0.3% H_2_O_2_ was added for 30 min to the tissue sections. Then, a citrate buffer solution was added to the tissues and heating was done in a microwave for 24 min. Then, a diluted solution of MYD88 rabbit mAB (1:200, A19082, ABclonal) was added. Immunostaining was performed utilizing Mouse/Rabbit ImmunoDetector DAB-HRP Brown Detection System (BSB 0003H, Santa Barbara, CA93117, United States of America). Staining of a negative control specimen was performed without adding the primary antibodies. Tissue sections were examined by a light microscope (CX23LEDRFS1, Evedent Corporation, Nagando Tokyo, Japan). Images were taken utilizing a calibrated digital camera. We performed image analysis procedures for estimation of the MYD88 immunostaining area % using ImageJ (NIH, United States of America) (Zaitone et al., 2026). The image analysis was done by an experienced physician in a blinded manner.

#### 2.4.7 Enzyme-linked immunosorbent assays (ELISA)

Spleen supernatants were used for the assessment of the following inflammatory biomarkers of TNF-α (SEA133Mu, sensitivity to less than 5.7 pg/mL) IL-1β (SEA563Mu, sensitivity to less than 5.5 pg/mL), NF-κB (MBS2023542, sensitivity is less than 0.057 ng/mL), MYD88 (MBS2022754, sensitivity is to less than 0.053 ng/mL), and TLR4 (MBS732114, sensitivity is 1 pg/mL) from MyBioSource and Cloud-clone Crope, United States of America. The ELISA kits were applied and readings were taken using an ELISA reader at wavelength value equals 450 nm. The assays were performed by a blinded experimenter. To reduce intra-assay variabilities, we used consistent pipettes, used uniform washing and volumes across all the plate wells, ensured adequate mixing of reagents and controlled the incubation periods.

### 2.5 Tabulation and data curation for statistical analysis

Data were collected, tablated and processed for statistical analysis. First, the normality of distribution of the quantitative data was confirmed by the Shaprio-Wilk test and then, analysis was performed by applying one-way ANOVA with a subsequent run of Tukey’s test. Second, a correlation analysis was done to check the correlation of the inflammatory markers to TLR4 by estimating the Pearson’s correlation coefficient with a 95% confidence intervals. We used the GraphPad Prism program for applying the tests.

## 3 Results

### 3.1 Explanation of TLR4-MYD88- NF-κB signaling pathways and its relevant genes

**FIGURE 2:**
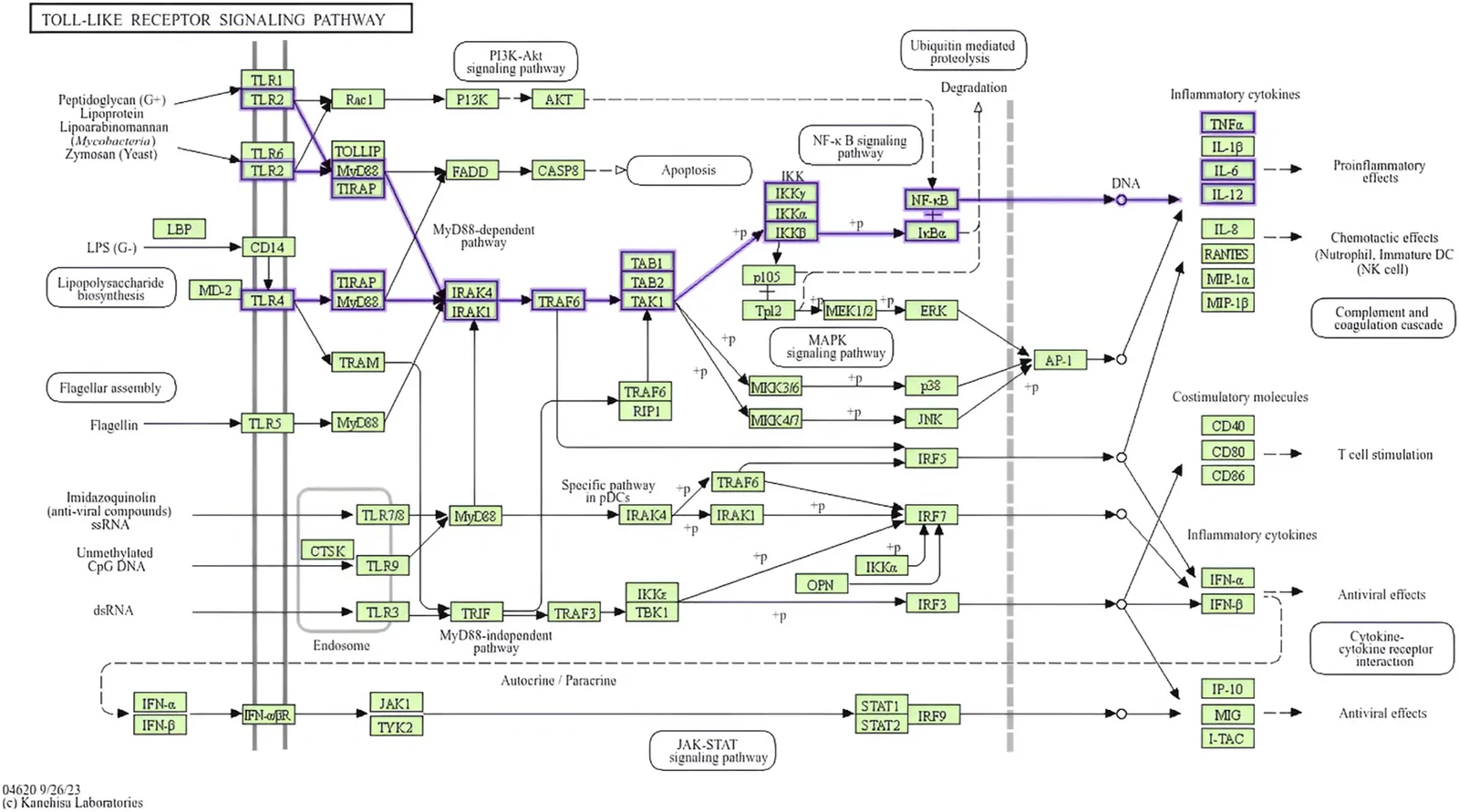
KEGG Toll-like receptor (TLR) signaling pathway highlighting TLR4-mediated immune activation and its subsequent pro-inflammatory effects. KEGG Copyright Permission (261230) by Kanehisa Laboratory (Kanehisa et al., 2023).

TLR signaling pathway (hsa04620) in KEGG database, describes the TLR interactions and visually explains the cascade of events upon stimulation and its relation with the downstream proteins (Figure 2). STRING analysis of the TLR4-centered signaling hub revealed over 100 protein interactions connecting it to pathways involved in cellular stress, apoptosis, cytokine release, and chemokine production. These genes were combined into the list with duplicates removal to determine if hesperetin’s targets any of those mediators.

### 3.2 The predicted targets of hesperetin and their relation to TLR4/MYD88/NF-κB signaling pathways

Swiss Target Prediction tool revealed 105 gene/protein targets highlighting a broad interaction profile for hesperetin. The Super-Pred dataset revealed 109 unique targets after exclusion of any targets with model accuracy less than 70%. PharmMapper analysis yielded 104 targets ranked by normalized fit score and limited to human targets only. The 3 lists were combined into one list which was manually curated followed by removal of duplicates and this resulted in a final list of 251 unique predicted targets for the studied bioflavonoid molecule. We selected TLR4 and its downstream signaling proteins as a putative target for hesperetin.

The diverse categories of the resulted genes suggest that hesperetin’s multi-target effects may hit several nodes simultaneously (receptor binding, kinase inhibition, ROS reduction, etc.) and could point to several possible interaction mechanisms with TLR4-MYD88-NF-κB pathway.

**FIGURE 3:**
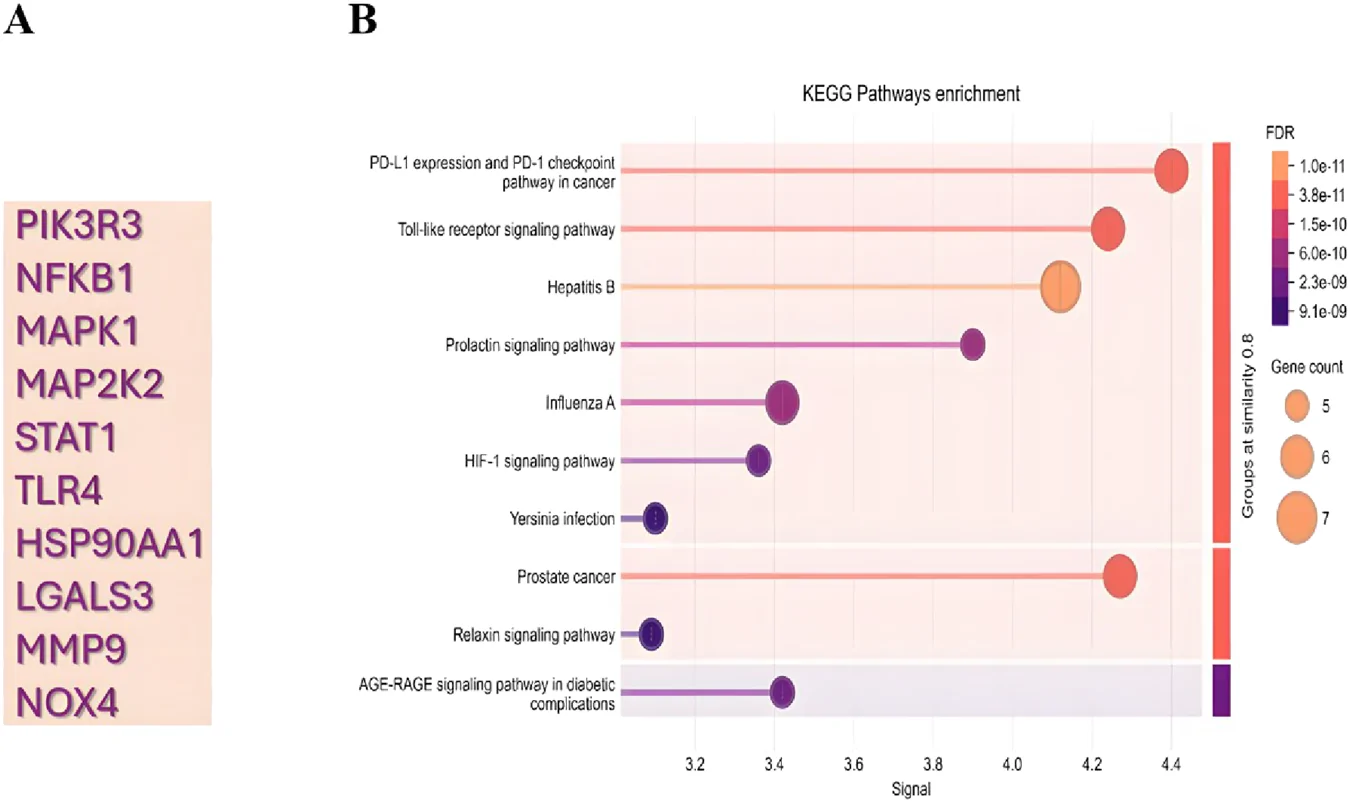
**(A)** List of ten intersecting genes of TLR–MYD88 pathway interactors that are simultaneously targeted by hesperetin. **(B)** Lollipop KEGG pathways enrichment chart of the top 10 pathways, color coded and sorted by fold enrichment. Chart was constructed using STRING db (FDR: false discovery rate).

The lists were intersected to pinpoint TLR–MYD88 pathway interactors that are simultaneously predicted to be targeted by hesperetin. Notably, ten intersecting genes emerged (Figure 3A), among them NF-κB1 and TLR4, the latter serving as a central hub with 8 direct connectors which intensified our rationale to select both for downstream experimental testing verification.

KEGG enrichment analysis via STRING database indicated that this gene set forms a central immune signaling hub, with highly significant enrichment (FDR < 1e-10) in TLR-associated pathways linked to infection, immunity, cancer, and inflammation. This confirms that hesperetin’s targets are genuinely concentrated in TLR4-mediated immune responses highlighting its broad therapeutic applicability and potential relevance to cancer treatment approaches (Figure 3B).

**FIGURE 4:**
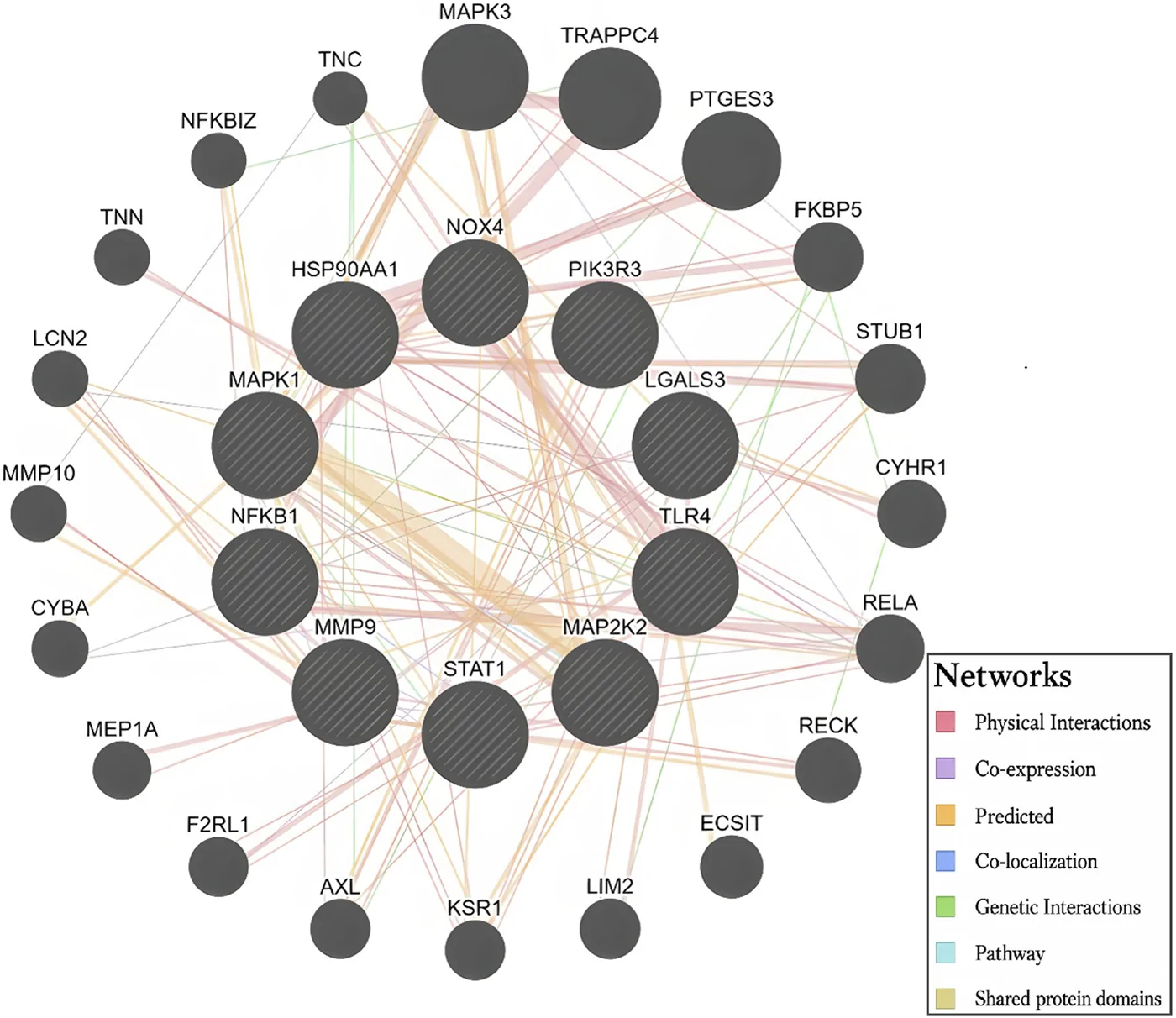
Semantic Web diagram showing the interacting genes targeted by hesperetin in relation to TLR4 pathway; 30 related genes with the 10 attributed inquiry genes (central circles indicated with stripes) and 151 total color-coded links with each color code, as detailed in the inset box, denoting a specific network interaction type. These intercalated links imply high average node degree and high clustering. Diagram was generated via GeneMANIA via automatically selected weighting method.

To gain biological insight into these interactions, this set of hub genes was imported to GeneMANIA (https://genemania.org/) to predict gene function, expand gene lists and construct interaction networks for biological interpretation and hypothesis generation for their interactions, co-localization, co-expression, and protein domain similarity. The analysis revealed that together they form a central inflammatory-stress module that integrates multiple pathways. The network is not a linear pathway but represents a densely interconnected web of pathways including TLR-mediated innate immune sensing, NF-κB driven transcriptional activation, inflammasome signaling, and ROS-dependent tissue injury as shown in (Figure 4).

### 3.3 Molecular docking

**FIGURE 5:**
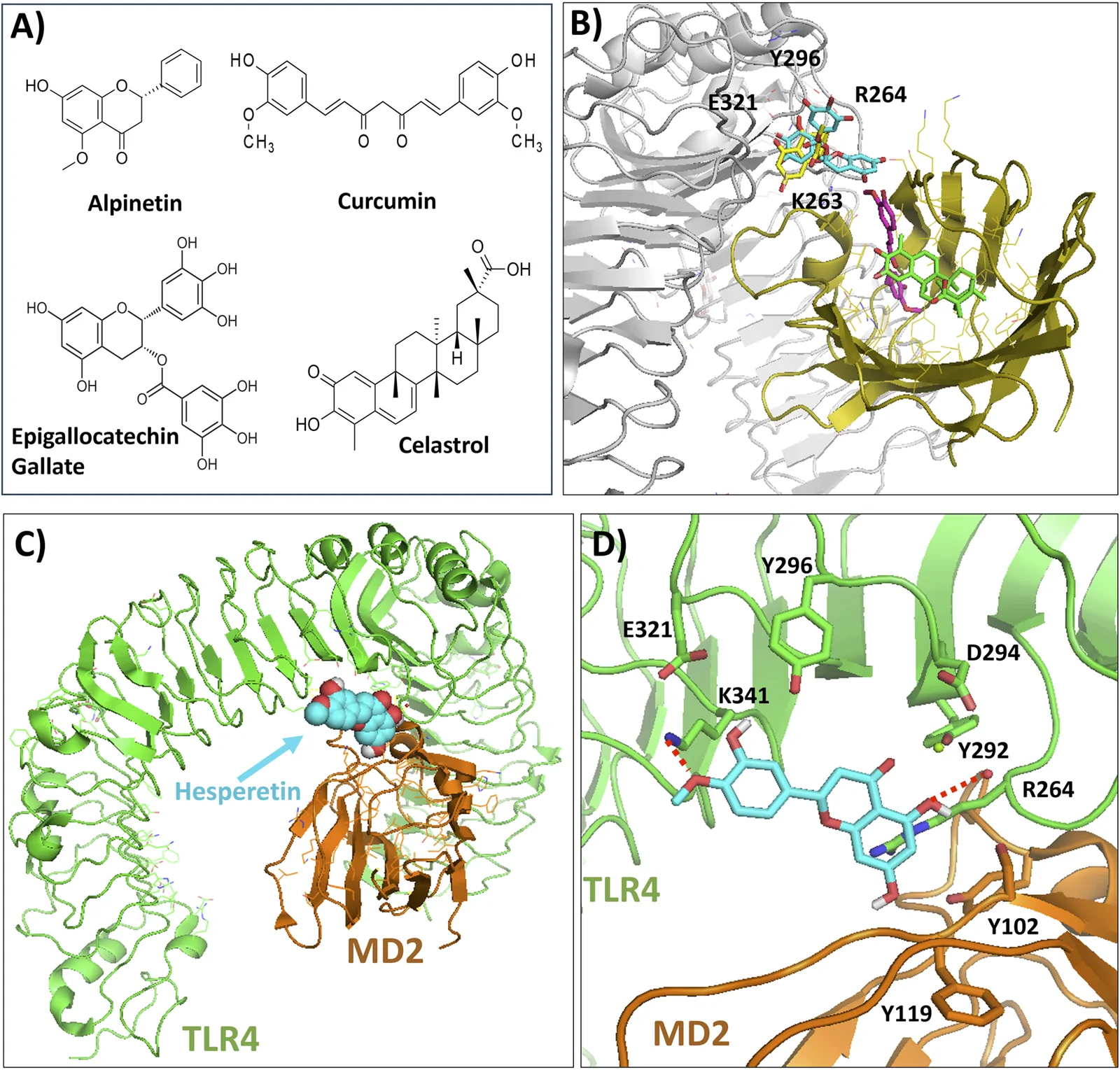
Proposed binding mode of hesperetin into the TLR4-MD2 interface. **(A)** Ligands used for the docking validation; **(B)** Cartoon representation of the TLR4 (grey)-MD2 (gold) interface, showing the docked ligands as sticks (alpinetin: yellow, epigallocatechin gallate: cyan, curcumin: purple, and celastrol: green); **(C)** Ternary complex of hesperetin-TLR4-MD2, showing the binding of hesperetin at the interface between the two proteins; **(D)** Cartoon representation of the binding site of hesperetin, composed of residues from TLR4 (green carbons) and from MD-2 (brown carbons). The ligand residues are represented with cyan carbons.

Our bioinformatics analysis highlighted TLR4 as a critical central hub, justifying its selection as the primary docking target. Prediction of any potential binding mode for hesperetin with TLR4, a docking study was performed for this ligand into the same interface exploited by the lipopolysaccharide at the protein dimer (PDB ID: 3FXI) of TLR4-MD2 (Park et al., 2009). We did not use any other constraints to control the orientation of the ligand inside this large interface. The hesperetin docking score was – 5.94 which is satisfactory compared to its size and to the score of the redocked lipopolysaccharide of – 14.895 (experimental section). Moreover, to further validate our docking approach, we docked selected small-molecule TLR4 inhibitors from natural sources (Figures 5A,B). These molecules showed comparable docking score to hesperetin (epigallocatechin gallate: −5.499, curcumin: −7.15, alpinetin: −5.04, celastrol: −5.98). As expected, it was also noticed that polar hydroxylated compounds such as the flavonoids alpinetin and epigallocatechin made H bonds contacts with the polar residues of TLR4 at the interface such as Arg264, Tyr296, Glu321, and Lys362. However, the more lipophilic molecules such as curcumin and celastrol mostly occupy the hydrophobic core of the MD2 ß-cup.

Then, we turned our attention to the investigation of the hesperetin binding mode. The best pose shows that the polar moieties of hesperetin binds with two of the residues which anchor the *E. coli* lipopolysaccharide, namely, Tyr296 and Lys341 (Figures 5C,D). These residues were also involved in the binding of the polar natural inhibitors of TLR4 as described above. It was noticed that the phenyl ring of hesperetin extends towards the polar network of TLR4 composed of several tyrosine and lysine residues, while the benzopyran scaffold reaches the entrance of the MD2 hydrophobic ß-cup. Also, one of the benzopyran OH groups shows a water-mediated H bond with Asp294 as shown by the *E. coli* lipopolysaccharide.

### 3.4 Effect of hesperetin on hematological parameters in vincristine treated mice

As presented in Table 1, vincristine affected the WBCs as it significantly diminished their blood count compared to the saline group mice. Concomitant administration of hesperetin with vincristine protected against the observed leukopenia and this group showed significant increment in WBCs count versus the vincristine group. The same effect was shown on the platelets count as vincristine reduced their count significantly, while co-administration of hesperetin retrieved their count to the saline control level. However, there were no significant effects on RBCs count and hemoglobin. Importantly, the hesperetin *per se* group did not show a significant change in the blood parameters compared to the saline group (Table 1).

**TABLE 1:** Effect of hesperetin on the hematological parameters in vincristine treated mice.

Hematological parameters	Saline control	Vincristine	Vincristine + HST	HST *per se*
WBCs (x 10^9^/L)	9.48 ± 0.16	4.6 ± 0.26 *	7.72 ± 0.12#	9.43 ± 0.14
RBCs (x 10^12^/L)	7.78 ± 0.17	7.63 ± 0.2	7.39 ± 0.33	7.89 ± 0.68
Hemoglobin (g/dL)	14.18 ± 0.36	12.96 ± 0.68	12.97 ± 0.87	13.36 ± 0.95
Platelets (x 10^9^/L)	1654.14 ± 194.26	799.14 ± 21.31*	1213.28 ± 106.23^#^	1646.14 ± 287.92

Data are demonstrated as mean ± SD).

* Versus control.

^#^ Versus vincristine group, p < 0.05.

### 3.5 Effect of hesperetin on histopathological alterations induced by vincristine

**FIGURE 6:**
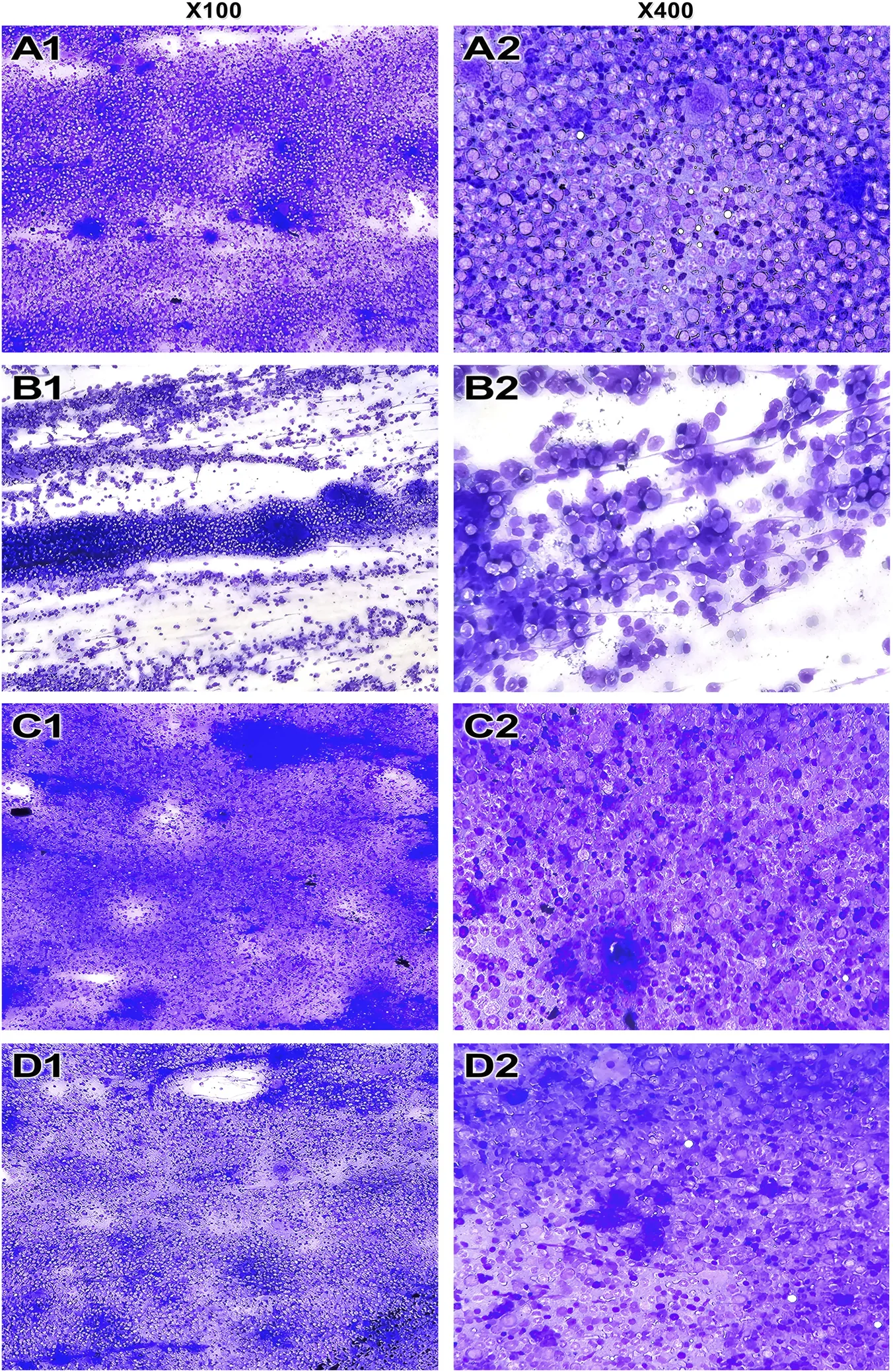
Effect of hesperetin on Giemsa histopathological alterations of bone marrow induced by vincristine. **(A1,A2)** The saline control group: bone marrow showed normal architecture with ∼95–100% cellularity, **(B1,B2)** vincristine group: the bone marrow demonstrates moderate-to-marked hypocellularity (∼30% cellularity), (**C1,C2)** the vincristine + hesperetin group: the bone marrow reveals an improvement in the marrow cellularity (∼80–90% cellularity). **(D1,D2)** The hesperetin *per se* group showed cellularity similar to the saline group.

Figure 6 shows staining of the bone marrow specimens with Giemsa stain. The saline group showed regular architecture with ∼95–100% cellularity, intact trilineage hematopoiesis, and a myeloid-to-erythroid (M:E) ratio of approximately 2.5:1. All granulocytic and monocytic lineage cells were included in the myeloid category for M:E ratio calculation (Figures 6A1,A2). While, the bone marrow of vincristine mice demonstrates moderate to marked hypocellularity (∼30–40%), with an M:E ratio of ∼1.5:1. Trilineage hypoplasia is evident, though the residual hematopoietic cells show normal morphology with preserved sequential maturation (Figures 6B1,B2). Sections from vincristine + hesperetin group reveals remarkable marrow response in comparison to vincristine group with total cellularity is approximately 80%–90%. The M:E ratio was equal to ∼2.5:1. Myeloid and erythroid expansion was observed (myeloid expansion was most pronounced). Trilineage morphology remains better than the vincristine group with preserved sequential maturation (Figures 6C1,C2). Importantly, the hesperetin *per se* group did not show a significant change in the bone marrow specimens compared to the saline group (Figures 6D1,D2).

**FIGURE 7:**
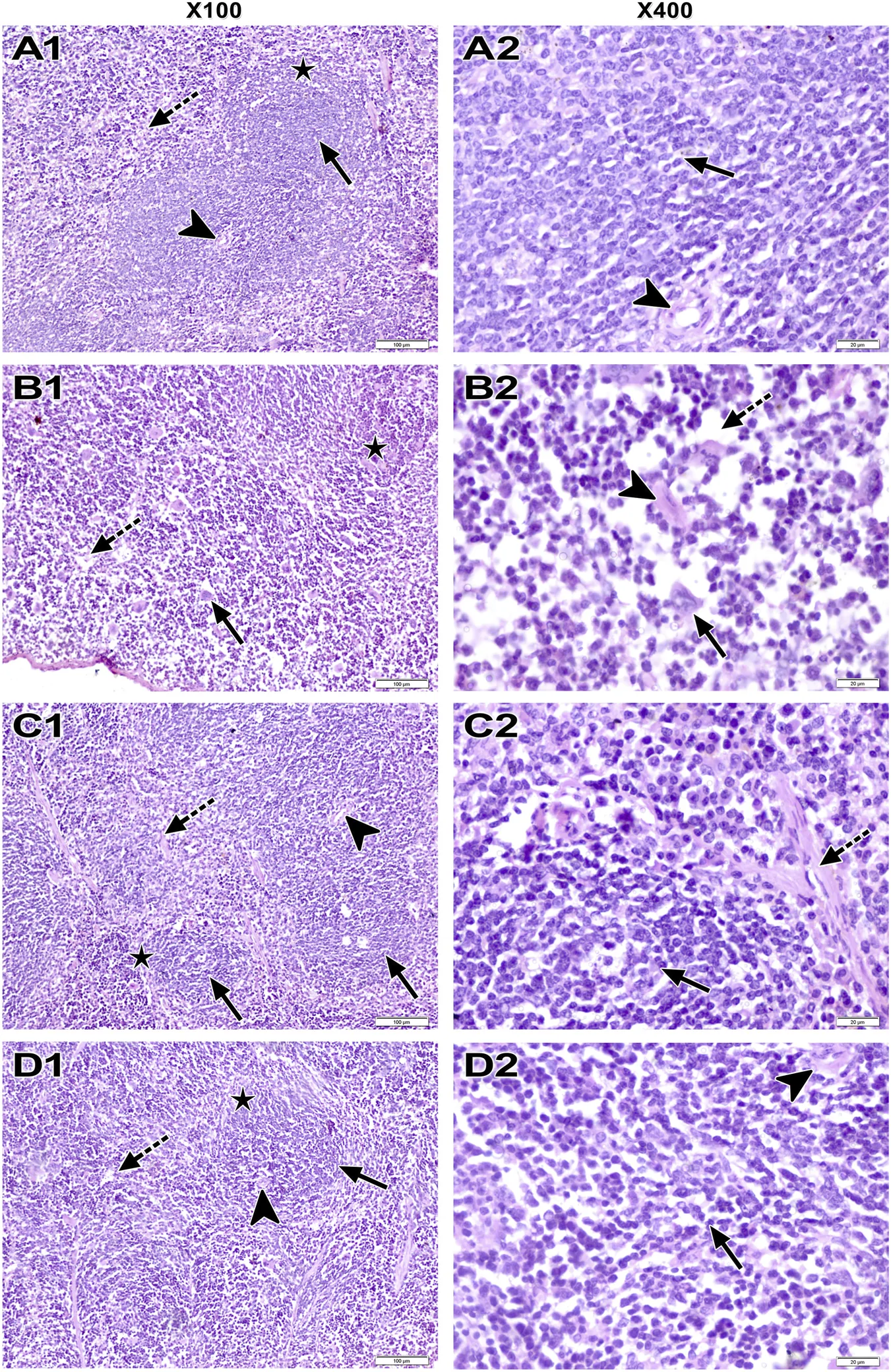
Effect of hesperetin on histopathological alterations of splenic tissues induced by vincristine. **(A1,A2)** The saline control group: images show normal splenic histoarchitecture. The white pulp lymphoid follicles are identifiable (arrow) with central arteriole of average wall thickness (arrowhead) with evident marginal zone (asterisk) while red pulps showed sinusoids (dashed arrow) and bounded by a thick fibrous capsule. **(B1,B2)** The vincristine group: splenic tissues show changes in architecture, effaced follicles showing shrinkage, thinning of the periarteriolar lymphoid tissue, septal hyalinosis (asterisk) and edema (dashed arrow). There is evidence of extramedullary hematopoiesis, in the form of megakaryocytes (arrow), arterioles are thickened and mostly narrowed to obliterated (arrowhead). **(C1,C2)** Vincristine + hesperetin group: the spleen specimens show both white and red pulp regions, with detectable lymphoid follicles (arrow) and average arteriolar wall (arrowhead). The contour of the follicle becomes better although shrunken center with prominent marginal zone (asterisk), and few prominent septa (dashed arrow) can be attributable to subcapsular location or proximity to hilum. **(D1,D2)** The hesperetin *per se* group showed histoarchitecture similar to the saline group.

Additionally, the splenic tissues stained with H&E is shown in Figure 7. The spleen exhibited normal histoarchitecture in the saline control mice, with red pulp and white pulp clearly demarcated and surrounded by a thick capsule. There were fibrous trabeculae shown extending into the splenic parenchyma. It was found that the white pulp encloses periarteriolar lymphoid sheaths. On the other hand, a central artery appeared in the lymphoid follicles with marginal zones were surrounding them. The red pulp appeared consisting of splenic cords disconnected from each other by splenic sinusoids (Figures 7A1,A2).

However, sections from vincristine group show an intact capsule. The white pulp demonstrates lymphoid depletion, evident as follicular shrinkage and thinning of the periarteriolar lymphoid sheath (Figures 7B1,B2). The red pulp reveals mild hypocellularity, sinusoidal congestion, and a relative increase in macrophages. The spleen specimens of vincristine + hesperetin group show remarkable expansion of both white and red pulp regions, with increased cellularity consistent with extramedullary hematopoiesis (Figures 7C1,C2). The hesperetin *per se* group did not show a significant change in the histological architecture of the spleens compared to the saline group (Figures 7D1,D2).

In the current study, scores given to splenic specimens stained with H&E staining are shown in Table 2. The scores for periarteriolar lymphoid sheaths cellularity, marginal zones lymphoid follicles, and red pulps for the experimental groups are individually shown and significant increases were highlighted in the vincristine group versus the saline group. Importantly, the total score in the vincristine group was greater than the saline group (15.86 ± 1.01 vs. 0, p < 0.05). The vincristine + hesperetin group showed significantly lower score versus the vincristine control group (7.71 ± 1.79 vs. 15.86 ± 1.01). Meanwhile, the hesperetin *per se* group showed pathology score equals zero.

**TABLE 2:** Effect of hesperetin on spleen histopathologic scoring in vincristine treated mice.

Comparison criteria	Saline	Vincristine	Vincristine + HST	HST *per se*
Cellularity of periarteriolar lymphoid sheaths	0	3.14 ± 0.69	1 ± 0.82	0
Lymphoid follicles	0	3.71 ± 0.49	2 ± 0.58	0
Marginal zone	0	3.85 ± 0.34	2.14 ± 0.69	0
Red pulp	0	2.71 ± 0.49	1.14 ± 0.69	0
Total number of germinal centers	0	2.43 ± 0.53	1.43 ± 0.53	0
Total score	0	15.86 ± 1.01*	7.71 ± 1.79^#^	0

Data are the average scores for each group and SD.

* versus the saline group.

^#^ Versus vincristine group at p < 0.05.

### 3.6 Effect of hesperetin on immunohistochemical staining changes of MYD88 induced by vincristine

**FIGURE 8:**
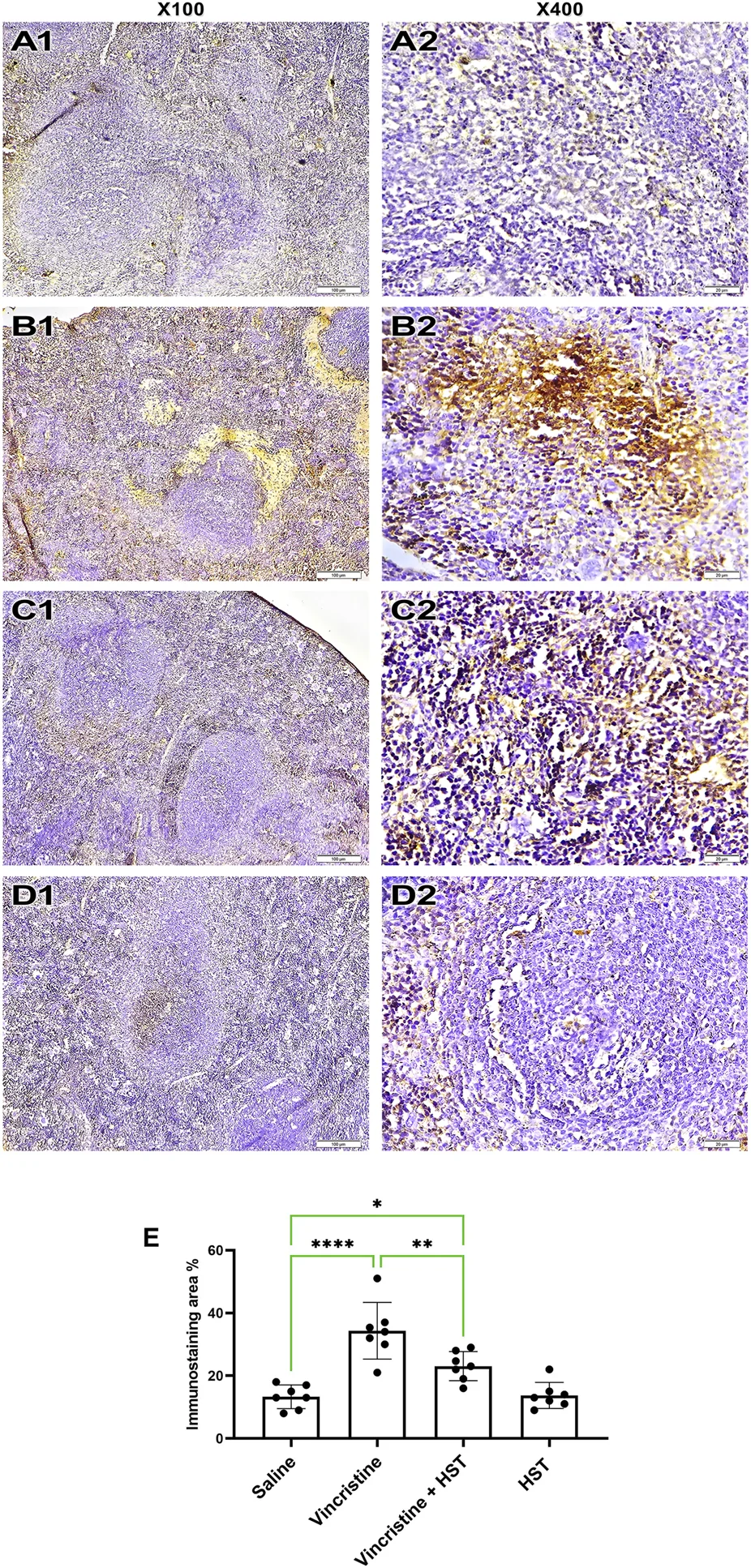
Effect of hesperetin on MYD88 immunohistochemical staining in specimens from mice spleens. **(A)** Saline control group: Images show a normal MYD88 immunostaining pattern. **(B)** Vincristine group: show marked increase of MYD88%, correlating with lymphoid depletion. **(C)** Vincristine + hesperetin group: the MYD88 staining was increased versus the vincristine group. **(D1,D2)** The hesperetin *per se* group showed immunostaining similar to the saline group. **(E)** Column charts for the area of immunostaining, data are mean ± SD. * versus the saline group, # versus vincristine group (P < 0.05).

As demonstrated in Figure 8; splenic tissues of the saline control mice illustrated normal MYD88 immunostaining pattern. The white pulp shows occasional MYD88-positive lymphocytes (∼13%) within periarteriolar lymphoid sheaths and follicles, consistent with physiologic low-level expression in B-cell zones. The red pulp and marginal zone demonstrate strong and diffuse MYD88 positivity, reflecting its normal expression in macrophages, monocytes, and dendritic cells, thereby maintaining intact splenic architecture (13.29 ± 3.77, Figures 8A1,A2). However, in the vincristine group, a markedly increased MYD88 area of staining is observed across all compartments, correlating with lymphoid depletion (34.33% ± 9.01%, Figures 8B1,B2). While, in the vincristine + hesperetin mice, MYD88 distribution pattern was decreased compared to vincristine group (23.1% ± 4.65%, Figures 8C1,C2). Finally, the vincristine *per se* group should a pattern of immunostaining closely resembling that observed in the saline group (13.71% ± 4.15%, Figures 8D1,D2). Figure 8E shows the mean area % of MYD88 immunostaining.

### 3.7 Hesperetin prevents the vincristine-induced increase in the level of the inflammatory biomarkers in the spleen

**FIGURE 9:**
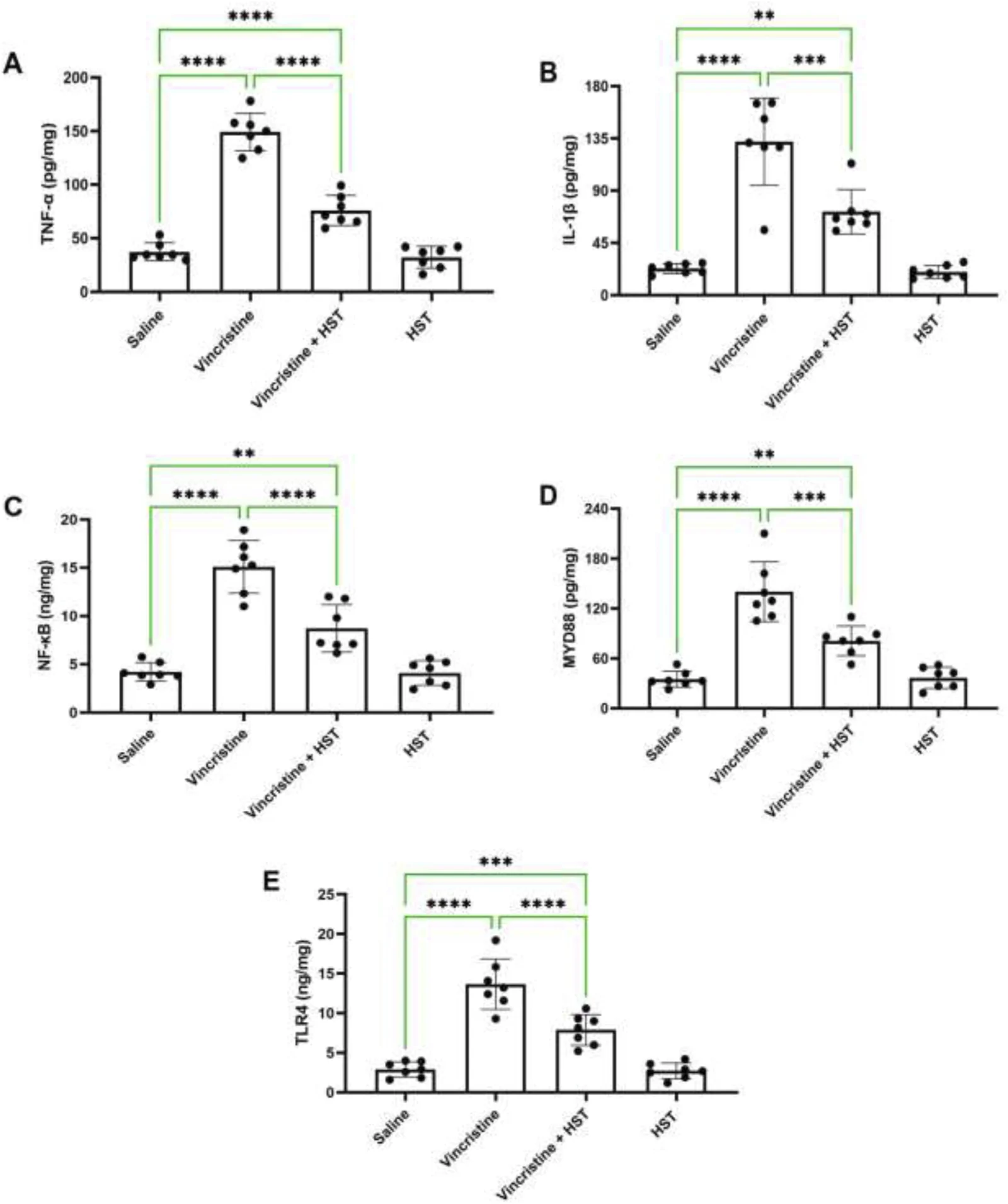
Effect of hesperetin on the vincristine induced target biomarkers in the spleen. Bar charts demonstrate inflammatory markers detected by ELISA in spleen supernatants of **(A)** TLR4, **(B)** NF-κB, **(C)** MYD88, and **(D)** TNF-α, and **(E)** IL-1β. Data are demonstrated as mean ± SD. * versus the saline group, # versus vincristine group (P < 0.05).

**FIGURE 10:**
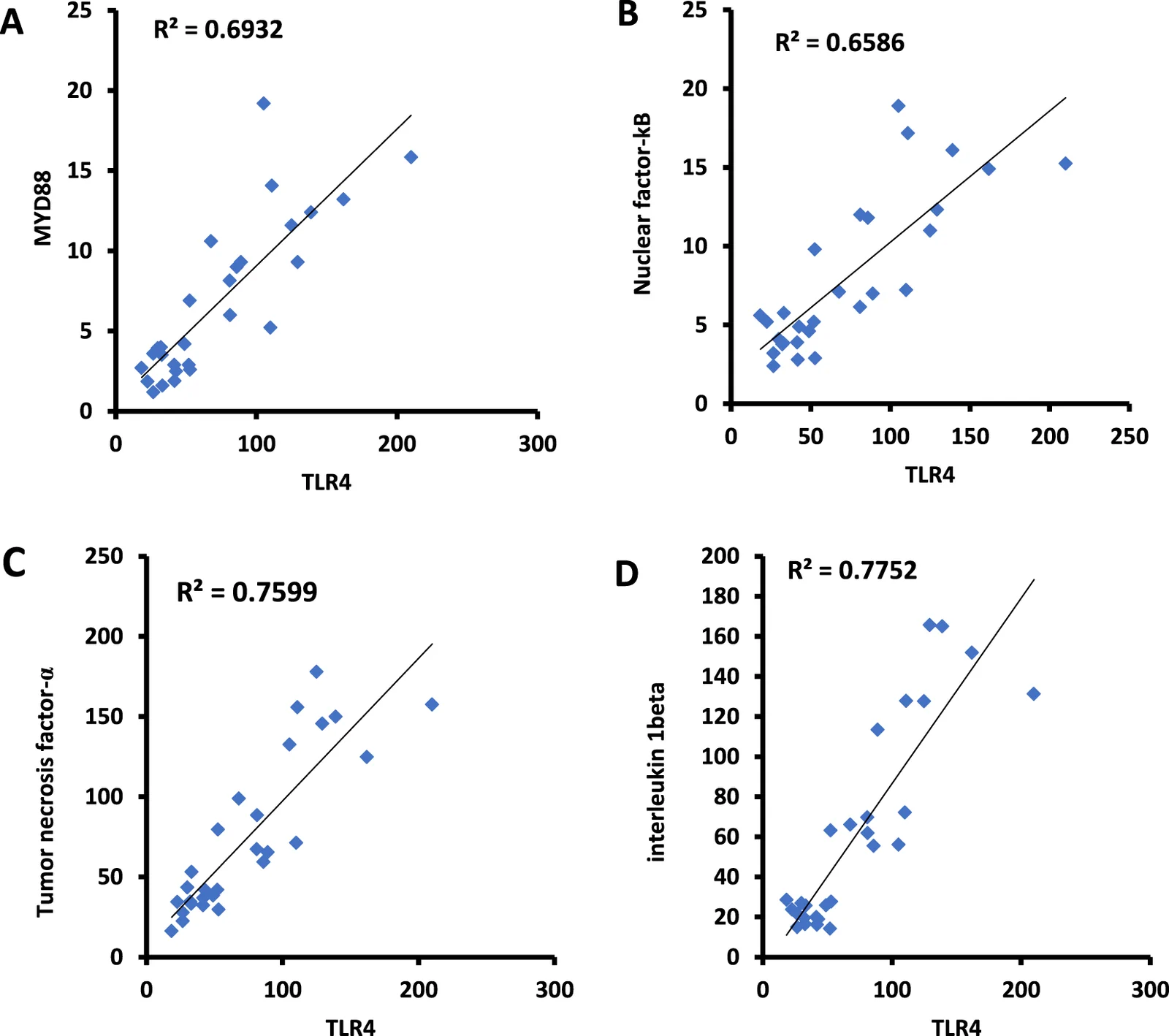
Correlation using Pearson’s correlation analysis between TLR4 and the other inflammatory markers. The correlation between TLR4 and **(A)** MYD88, **(B)** NF-κB, **(C)** TNF-α and **(D)** IL-1β are shown.

Figures 9A–E illustrated that spleen content of TLR4, MYD88, NF-κB, TNF-α and IL-1β were found markedly upregulated in the vincristine group versus the saline group. However, vincristine + hesperetin group showed downregulated TLR4, NF-κB, MYD88, TNF-α and IL-1β significantly as compared to vincristine group. The hesperetin *per se* group did not show any significant difference in these markers versus the saline group. Figures 10A–D illustrate moderate-to-strong positive correlations between spleen content of TLR4 and MYD88 (*r* = 0.83), NF-κB (*r* = 0.81), TNF-α (*r* = 0.87), and IL-1β (*r* = 0.88).

## 4 Discussion

Vincristine is classified as a Vinca alkaloid chemotherapeutic agent which produces hematological toxicity especially at high or cumulative doses (Kosmidis et al., 1991; Awosika et al., 2023). However, in case of vincristine overdose, myelosuppression can occur in animals but neurotoxicity remain the primary concern side effect (Rubio et al., 2004; Musser et al., 2021). Notably, vincristine induces mild to moderate hematological suppression, particularly impacting platelet and leukocyte counts. However, myelotoxicity resolves upon dosage modification or its cessation, severe cases may require supportive interventions including blood product transfusions or hematopoietic growth factor therapy (Chae et al., 1998). This agrees with the findings of the current study which demonstrated by CBC analysis that the WBCs and platelet counts were found declined but the other blood components (RBCs and hemoglobin) were not affected significantly.

Several mechanisms and interventions were proposed to limit vincristine’s myelotoxicity and extramedullary hematopoiesis by the spleen. In the current study, vincristine treated group showed marked increase of spleen content of TLR4, MYD88, and NF-κB with consequently upregulated TNF-α as well as IL-1β content in splenic tissues. Thus, these findings came in line with previous studies (Rajput et al., 2013; Ran, 2015) as this studies supposed that TLR4 activation contributes to chemotherapy-induced inflammation and metastasis. Guven-Maiorov et al. (2015) showed that the signaling cascade initiated by TLR activation influences virtually every stage of cancer development, from initial tumor formation to progression and metastasis (Guven-Maiorov et al., 2015). Moreover, Son et al (2019) showed that treatment with the chemotherapeutic agent paclitaxel -which is a TLR4 agonist-induces TLR4-dependent phosphorylation of JNK and IκB, increases proinflammatory cytokine mRNA, and releases IL-1β in macrophages as well as splenic hematopoietic cells, highlighting a mechanism for chemotherapy-driven myelosuppression (Son et al., 2019). Furthermore, vincristine was reported to stimulate the canonical NLRP3 inflammasome in macrophages, promoting IL-1β secretion involved in neuro-inflammation mediated vincristine-induced peripheral neuropathy (Starobova et al., 2021).

Vincristine myelotoxicity manifested herein as leukopenia and thrombocytopenia may be attributed to its binding to tubulin that causing microtubule depolymerization. This not only interferes with mitotic spindle formation but also stimulates the TLR4 signalosome, since receptor clustering leads to endosomal trafficking (Guven-Maiorov et al., 2015).

The bioinformatic study showed the TLR signaling pathway in KEGG database, describing the TLR interactions and its relation with the downstream proteins. TLR4 was shown to be a major pattern recognition receptor that interacts with various key proteins, one of which is MYD88 which is an adaptor protein that bridges TLR4 and IL-1R to downstream signaling kinases. Following a cascade of events, the pathway eventually leads to production of NF-κB, which is the master transcription factor that controls a canonical inflammatory cascade and various immune genes as TNF-α, IL-1β and IL-6. Cytokines are known as strong inducers of apoptosis in CD34^+^ hematopoietic progenitor stem cells and result in loss of bone marrow cellularity. Furthermore, cytokines suppress erythropoietin-mediated erythropoiesis which ultimately drives the exhaustion of those progenitor cells. Thus, blocking or hindering such pathway would result in amelioration of this myelotoxic effect.

Consequently, the MYD88 adaptor molecule, causes activation of IκB kinase activity and induced NF-κB nuclear translocation (He et al., 2024). By association with activating MYD88-mediated NF-κB signaling, vincristine stimulates pro-inflammatory cytokines transcription genes. This mechanism links its microtubule-targeted action to activation of TLR4-driven inflammatory downstream in hematopoietic cells which contributes to its cytotoxicity (Bian et al., 2017). In the current study, the correlation analysis supports the relationship between the studied signaling proteins.

The GeneMANIA analysis revealed a central inflammatory-stress module that integrates multiple pathways. A non-linear pathway was shown including TLR-mediated innate immune sensing, NF-κB driven transcriptional activation, inflammasome signaling, and ROS-dependent tissue injury. This implies that if hesperetin blocks any single node, it will affect multiple downstream processes as they are functionally coherent.

TLR4 is a critical central hub, justifying its selection as the primary docking target. This transmembrane receptor is characterized by three distinct regions: an external leucine-rich repeat, a transmembrane bridge, and an internal Toll/IL-1 receptor domain. When ligands such as lipopolysaccharide dock with the outer leucine-rich repeat, the receptor is activated, facilitating the transmission of downstream signals through the Toll/IL-1 receptor domain. Structural data from PDB ID: 3FXI indicates that ternary complex of TLR4/MD2/lipopolysaccharide is configured in M-shaped dimer consisting of a couple of TLR4/MD2 pairings (Park et al., 2009). In this configuration, the beta-cup protein MD2 provides a large hydrophobic cavity that encapsulates five out of the identified six aliphatic lipopolysaccharide chains, while the remaining chain resides on the surface. Stabilizing the interface within TLR4 and MD2 is maintained by an intricate network of polar interactions involving the lipopolysaccharide phosphate head groups and specific residues, namely, Lys263, Gln339, Lys341, and Lys360 belonging to TLR4, along with Ser120, Tyr102, and Glu122 belonging to MD2.

It was noticed that hesperetin docks to TLR4 and we believe that hydroxylated flavan-4-one nuclei possess a good complementary shape with the molecular interface of TLR4/MD2 protein, and were able to make an intricate polar contact network with the residues. However, the benzopyran nucleus was found to partially fill the MD2 internal ß-cub hydrophobic pocket. We can envision that attaching one or more lipophilic moieties to its hydroxyl groups would impart higher affinity to the ligand, and could be used as a starting point for the design of clinically useful candidates.

Hesperetin, is a naturally occurring flavanone, it can be found abundantly in citrus peels, exerts strong antioxidant effects and activation of cellular defense pathways (Mirzaei et al., 2023). It is known also to deliver its anti-inflammatory benefits by inhibiting NF-κB signaling (Ren et al., 2016). Moreover, leveraging hesperetin’s multifaceted modulation of the inflammatory cascade holds promise as a strategy to prevent or mitigate drug-induced toxicities (Khan et al., 2020; Ji et al., 2024).

Several findings reveal hesperetin’s multi-targeted activity, it scavenges reactive oxygen species, disrupts upstream receptor-mediated TLR4/NF-κB signaling pathways (Muhammad et al., 2019), that orchestrate the inflammatory response, abrogated influx of inflammatory cells (neutrophils, eosinophils, macrophages, and lymphocytes), and lowers key cytokines of TNF-α (IL)-2, −4, and −5, and interferon-γ (Shih et al., 2012). At the molecular level, hesperetin attenuates inflammation by modulating key signaling pathways “NF-κB”, promotes IκB-degradation and suppresses the expression of cyclooxygenase-2 and iNOS. These findings underscore hesperetin’s multi-targeted approach: it not only scavenges reactive oxygen species but also disrupts upstream receptor-mediated signaling cascades and key transcription factors that orchestrate the inflammatory response (Ren et al., 2016).

Herein, hesperetin treatment prevented the increases in the spleen content of TLR4, MYD88 and NF-κB of vincristine-treated mice along with significant decreases in the inflammatory markers (TNF-α and IL-1β). These results came in the same line with Lee et al. (2021) who showed that hesperetin downregulates TLR/MYD88/NF-κB signaling, resulting in decreased production of pro-inflammatory molecules in human THP-1 monocytes exposed to lipopolysaccharide and high glucose (Lee et al., 2021). Hesperetin strongly validates as a promising natural bioactive molecule for inflammatory disease therapy and prevention in experimental animals (Shree et al., 2022).

Research reveals that hesperetin exhibits selective and potent inhibitory capabilities against key inflammatory enzymes and mediators. Specifically, it effectively suppresses critical inflammatory signaling pathways by modulating TLR4/NF-κB cascades, reducing-the manufacture of the inflammatory cytokines, and inhibiting inflammatory enzymes (Muhammad et al., 2019).

In previous studies and animal models, hesperetin has been shown to offer neuroprotection via mitigating Nrf2/TLR4/NF-κB signaling (Ikram et al., 2019) or inhibition of NF-κB phosphorylation and Nrf2/HO-1 activation (Ren et al., 2016) together with its potent anti-apoptotic effect. Moreover, such a neuroprotective effect is intently related to its strong inhibition of oxidative stress, neuroinflammation, and cognitive consolidation (Spagnuolo et al., 2018; Stephenson et al., 2018). One recent experimental study highlighted that oral administration of hesperetin offers a protective inflammatory control; it acted as a prebiotic that regulated the composition of the intestinal flora via controlling the levels of short-chain fatty acids, tightening of the epithelial junction, and reducing inflammation through preserving the TLR4/NF-κB axis in the mouse model (Ran et al., 2024).

A number of breast cancer patients highly express TLR4 (Mai et al., 2013; Volk-Draper et al., 2014). While inhibition of TLR4/NF-κB signaling was reported to provide cytotoxicity and antitumor activity in breast cancer cell line (Zandi et al., 2020). In the same context, studies on hepatocellular carcinoma have reported that over-activation of the TLR4/MYD88/NF-κB pathway may cause dysfunction in immune response, leading to tumorigenesis (Chen et al., 2018; Lupi et al., 2020; Hassan et al., 2021). Hence, inhibiting of this signaling pathway may help suppressing tumor initiation and progression (Vandewynckel et al., 2014; Wu et al., 2025). Similarly, a colorectal carcinogenesis model indicated that blocking of MYD88 signaling can disrupt secretion tumor/inflammation-derived factor (Wang et al., 2022) while transgenic blocking of MYD88 signaling attenuates lung and ovarian tumors growth (Hong et al., 2013; Baert et al., 2019).

While the suppression of TLR4/MYD88/NF-κB signaling offers clear benefits in mitigating chemotherapy-induced inflammation and myelosuppression, it is important to acknowledge the dual role of this pathway and the potential risks associated with its inhibition. The TLR4/MYD88/NF-κB axis serves as a critical component of innate immune defense, mediating pathogen recognition and initiating antimicrobial responses (Mogensen, 2009; Kawai and Akira, 2010). Complete or excessive suppression of this pathway may increase susceptibility to opportunistic infections, particularly in immunocompromised cancer patients undergoing chemotherapy (Hennessy et al., 2010). Furthermore, impaired TLR4 signaling can compromise tissue repair and regeneration processes, as NF-κB activation is essential for coordinating inflammatory resolution and wound healing responses (Lawrence, 2009).

Paradoxically, while chronic NF-κB activation promotes tumor progression, this pathway also plays important roles in antitumor immunity, including activating natural killer cells, and dendritic cell maturation (Karin and Greten, 2005; Karin, 2006). Therefore, complete ablation of NF-κB signaling could potentially diminish antitumor immune surveillance and reduce the efficacy of certain immunotherapeutic strategies (Hayden and Ghosh, 2012).

Indeed, several studies reported that NF-κB has dual-function nature; although NF-κB activation supported cancer cell survival proliferation, metastasis, and angiogenesis, particularly in hematologic malignancies (Enzler et al., 2011; Lukas et al., 2025; Mao et al., 2025). Thus, refers the challenge to target NF-κB and its downstream targets as a therapeutic strategy and more investigations are needed to focus on molecular target-based therapy or cell-selective inhibition of NF-κB signaling to improve therapeutic index of chemotherapeutic agents and decrease host toxicity.

In this context, the protective effects of hesperetin observed in the current study may be attributed to its modulatory rather than complete suppressive action on the TLR4/MYD88/NF-κB pathway. Unlike complete pathway inhibitors, hesperetin appears to attenuate excessive inflammatory signaling while preserving basal immune function, as evidenced by the partial rather than complete reduction in inflammatory markers and the maintenance of immune cell populations in treated animals (Ren et al., 2016; Lee et al., 2021). This balanced modulation may represent a therapeutic advantage, allowing for the mitigation of chemotherapy-induced inflammation without compromising essential immune defense mechanisms or antitumor immunity. It is recommended to examine the ideal doses and timing of administering hesperetin for maximizing its protective benefits while lessening the risks associated with immune suppression.

This study investigated the effect of hesperetin (100 mg/kg) in a hesperetin *per se* group in healthy mice to eliminate any harmful effects for hesperetin alone. Importantly, one previous study investigated in mice the effect of higher oral doses of hesperetin (400 mg/kg) than the current dose (100 mg/kg) and did not document any significant effect on several functions and inflammatory markers (Bai et al., 2017). Furthermore, a preclinical investigation on the safety of a hesperetin derivative reported no toxic effects. Authors demonstrated that hesperetin exhibits high safety profile with minimal adverse effects establishing its viability for potential clinical applications (Moriwaki et al., 2023).

## 5 Conclusion

Collectively, hesperetin represents a promising natural agent for co-administration of chemotherapeutic agent protocols due to its favorable pharmacokinetic properties and broad modulation of inflammatory signaling networks. Hence, the present study proposes that hesperetin may be an effective co-treatment strategy to prevent vincristine-induced myelotoxicity.

As no previous studies were done to explore the effect of hesperetin on the anticancer effect of vincristine in tumor-bearing models, further studies are needed to test if hesperetin might interfere with vincristine’s anticancer effects; this is critical for any proposed co-treatment strategy. Further, the current study is a single-sex and single dose study which did not identify the dose-response relationships for hesperetin. Further, studies may be warranted to fully investigate the therapeutic effect of hesperetin.

The dose of hesperetin is 100 mg/kg, so according to the equation for dose translation between mouse and human (Reagan-Shaw et al., 2008) (mouse Km = 3, human Km = 37), the human equivalent dose (HED) is approximately 8.1 mg/kg (486.48 mg for a 60 kg adult), which is in the range of doses utilized in clinical studies for hesperetin and hesperidin flavanones (Rizza et al., 2011; Rangel-Huerta et al., 2015). However, further studies are warranted for dose translation from pre-clinical to clinical studies.

The limitations of this study include the short treatment duration (2 weeks) and the lack of mechanistic validation experiments (e.g., TLR4 knockout or using specific TLR4 inhibitors). Hence, we suggest follow-up studies with genetic or pharmacological TLR4 inhibition to validate mechanism and conducting a time-course study for understanding optimal treatment timing. Limitations also include the need for further validation by other complementary approaches such as Western blotting.

## Data Availability

The raw data supporting the conclusions of this article will be made available by the authors, without undue reservation.
